# The Use of Angiotensin-I Converting Enzyme I/D Genetic Polymorphism as a Biomarker of Athletic Performance in Humans 

**DOI:** 10.3390/bios2040396

**Published:** 2012-10-09

**Authors:** Maria Fernanda De Mello Costa, Ron Slocombe

**Affiliations:** 1 Waikato Institute of Technology, Tristam Street, Private Bag 3036, Waikato Mail Centre, Hamilton 3240, New Zealand; 2 Faculty of Veterinary Science, The University of Melbourne, 250 Princes Highway, Werribee 3030, VIC, Australia; E-Mail: r.slocombe@unimelb.edu.au

**Keywords:** Angiotensin I Converting Enzyme, athletic performance, biomarker

## Abstract

Angiotensin II is a key regulator of blood pressure and cardiovascular function in mammals. The conversion of angiotensin into its active form is carried out by Angiotensin I-Converting Enzyme (ACE). The measurement of ACE concentration in plasma or serum, its enzymatic activity, and the correlation between an insertion/deletion (I/D) genetic polymorphism of the ACE gene have been investigated as possible indicators of superior athletic performance in humans. In this context, other indicators of superior adaptation to exercise resulting in better athletic performance (such as ventricular hypertrophy, VO_2_ max, and competition results) were mostly used to study the association between ACE I/D polymorphism and improved performance. Despite the fact that the existing literature presents little consensus, there is sufficient scientific evidence to warrant further investigation on the usage of ACE activity and the I/D ACE gene polymorphism as biomarkers of superior athletic performance in humans of specific ethnicities or in athletes involved in certain sports. In this sense, a biomarker would be a substance or genetic component that could be measured to provide a degree of certainty, or an indication, of the presence of a certain trait or characteristic that would be beneficial to the athlete’s performance. Difficulties in interpreting and comparing the results of scientific research on the topic arise from dissimilar protocols and variation in study design. This review aims to investigate the current literature on the use of ACE I/D polymorphism as a biomarker of performance in humans through the comparison of scientific publications.

## 1. Introduction

The aim of this review is to discuss the current applications of the ACE I/D genetic polymorphism as an indicator that individual athletes possess characteristics which are advantageous from the perspective of achieving better athletic results, or better training outcomes.

In this context, the word “biomarker” is utilized to indicate a substance, its activity (in the case of enzymes), or a genetic characteristic which provides an indication that an individual possesses certain characteristics. Specifically, that ACE concentration, activity, or genetic polymorphism can be used as a form of identification of individuals with improved athletic ability, or who achieve better results in certain athletic competitions. 

In regard to the term “superior athletic performance” some explanation is required. Due to the variety of methods utilized in the papers included in this review, this term is loosely applied in the text as an indication that individuals demonstrated improved results during exercise training and/or athletic competition. Parameters utilized in the assessment of this improvement include well established variables such as ventricular hypertrophy as a result of controlled exercise, VO_2_ max, competition results, heart rate, standardised exercise testing, and time to recovery after exercise.

Genetic polymorphism: Although several genetic polymorphisms for the ACE gene have been identified in humans, the insertion (I)/deletion (D) polymorphism of a 250 base pair *alu* fragment on intron 16 of the ACE gene, which is located on chromosome 17, accounts for 47% of the variation in ACE activity in circulation [1]. Therefore the focus of this review is on this particular I/D polymorphism in humans, although two other polymorphisms are briefly mentioned.

## 2. Materials and Methods

In preparation for a Doctoral thesis [2], articles found on the University of Melbourne search engine associated with the keywords Angiotensin Converting Enzyme and athletic performance; Angiotensin Converting Enzyme and genetic polymorphism and athlete, dating from 1978 to 2011 were initially acquired for inspection. The University of Melbourne search engine includes databases such as *Scopus*, *Medline (ISI)*, *and CAB.* Reading of the abstracts of approximately 3,000 journal articles returned from the initial search led to the separation of 551 articles of some relevance to the topic of ACE as an indicator of superior athletic activity in various forms. In the preparation of this review, only articles directly related to the use of the ACE I/D genetic polymorphism as a biomarker of athletic performance in humans, dating from 1997 to 2011 were included. Older references were included in the section detailing the renin angiotensin system.

## 3. The Renin-Angiotensin System

The main interest in ACE arose from the fact that its activity was easily determined from blood samples and that variations from expected levels were found in certain diseases. The other major source of interest was the fact that certain drugs could inhibit the action of ACE, helping modulate the vasopressor response originating from the production of ANG II and aiding in controlling hypertension. More recently ACE activity has been demonstrated to correlate to genetic components and to athletic aptitude, which enlarged the clinical importance of ACE as a biomarker for performance prediction [3,4,5,6].

The Renin-Angiotensin System (RAS) is a peptidergic system that possesses endocrine characteristics. Renin is secreted by juxtaglomerular cells of the kidneys, mostly in response to reduced renal blood flow. Sodium depletion and direct adrenergic stimulation can also produce renin release [7]. 

Renin is a protease responsible for cleavage of the tetradecapeptide angiotensinogen, produced in the liver, into the active Angiotensin I (ANG I) [7,8]. Cleavage of Angiotensinogen occurs at the Leucine^10^-Valine^11^ in humans [9]. Once Angiotensin I is formed, it is converted into Angiotensin II (ANG II) mainly in the lung circulation [8,10] but also in other vascular beds [7]. The enzyme responsible for conversion of ANG I into ANG II is Angiotensin I-converting enzyme (ACE). 

Both ANG I and ANG II have vasoconstrictive properties and are involved in the control of mean arterial pressure both at rest and during exercise [10], although ANG II is more potent than its precursor. Angiotensin II, in conjunction with potassium levels, stimulates production and release of aldosterone and stimulates thirst and drinking reflexes [10]. Angiotensin II activates specific receptors in the vascular smooth muscle causing release of calcium from the sarcoplasmic reticulum. This calcium binds to calmodulin and causes vasoconstriction through contraction of vascular smooth muscle [7]. 

## 4. ACE Genetic Polymorphism and Performance in Humans

The characteristics of the ACE gene and the insertion/deletion polymorphism on intron 16 were described in the early nineties [2,11,12] but it was only in 1997 that a possible correlation between ACE I/D polymorphism, ACE activity in blood and factors affecting athletic performance was suggested [3]. Based on previous knowledge that the RAS regulates left ventricular growth, Montgomery and his collaborators [4] decided to test the association between ACE genetic polymorphism and improved athletic performance [3,5,6]. Since these first publications, several more addressed the issue of a possible association between ACE and individual characteristics which could result in better results from exercise training or better performance in athletic competitions [3,4,5,6,13,14,15,16,17]. The literature review suggests, although not unanimously, that athletes who present the ACE DD genotype, and therefore higher ACE activity in blood, demonstrate better results in sports which require speed, such as sprint running. On the other hand, individuals with the II genotype, and therefore a lower ACE activity in blood, demonstrate better results in sports requiring endurance, such as cross-country skiing, and mountaineering. However, there is impressive variation in the populations of athletes studied, their training regimes, and the sports practiced.

What seems to emerge from the publications is that certain ethnicities appear to demonstrate a stronger association between ACE activity or I/D polymorphism and athletic results. It also seems evident that individuals participating in certain endurance sports demonstrate more benefit from having the II genotype in comparison to other endurance sports. The key to the problem seems to be the separate investigation of ethnicities and sports, utilizing measurements of performance that can be utilized regardless of the type of training instituted.

Table 1 summarizes the work conducted by research groups which focused on the measurement of ACE I/D polymorphism in association with ventricular hypertrophy, the latter being a measurement of appropriate response to training.

**Table 1:** Summary of publications addressing the association between the I/D ACE polymorphism and ventricular hypertrophy as an indication of improved athletic ability and/or training in a variety of athletic modalities, athlete quality and ethnicities. Table adapted from previous work by the authors [1].

Reference	Number of subjects	Training/sport	Variable assessed as indicator of superior athletic ability	Measurements remarks	Findings	Special training protocol
[4]	308 Caucasian soldiers	10 weeks military training	left ventricular hypertrophy	pre-training *versus* post-training	DD genotype associated with left ventricular hypertrophy post training	no
[18]	80 Finish athletes	4 different sport modalities with different training regimes	left ventricular hypertrophy and ventricular mass	compared a sedentary group with the athletes in a single measurement	No association between ACE I/D polymorphism and ventricular hypertrophy	no
[14]	43 runners	ultra-marathon	left ventricular hypertrophy	single measurement	D allele associated with greater left ventricular hypertrophy	no
[13]	28 professional footballers	football	left ventricular hypertrophy	pre-training *versus* post-training	D allele associated with greater left ventricular hypertrophy	yes

Three of the four studies found a positive association between the presence of the D allele (or the DD genotype) and greater ventricular hypertrophy in response to exercise, despite the variation in sport, and training regimes. Only one of the studies [13] had a training protocol designed specifically to test improvement in fitness during the experiment, while the others [4,14,18] utilized whatever training the athletes were being subjected to, as part of their routine. 

The study utilizing Finish athletes [18], did not find an association between the I/D polymorphism and ventricular hypertrophy, however they found that another polymorphism (M235T) in the ACE gene actually correlates to greater ventricular hypertrophy. This was however the only published study focusing on the M235T polymorphism.

Other studies investigated the association between ACE and other indicators of improved athletic ability, such as VO_2_ max, and observed no differences between the distribution of ACE genotypes in endurance athletes in comparison to sedentary subjects [19]. However, the same research group has included the ACE gene as a potential source of physical characteristics of interest in athletes [20,21,22]. A different ACE polymorphism (T allele for the ACE T-3892C) was found to be associated with an improvement in the VO_2_ max of Korean women submitted to 12 weeks of endurance training [23], although the I/D polymorphism was not investigated. It is very unlikely that a single genetic polymorphism would be positively associated to all established measurements of superior athletic ability, which does not invalidate the use of the I/D polymorphism as an indicator of the degree of cardiac hypertrophy in response to exercise.

A summary of publications investigating the use of ACE I/D genetic polymorphism as a predictor of superior athletic performance in high calibre athletes can be found in Table 2. 

**Table 2:** Summary of studies that focused on performance results and its association with the ACE I/D polymorphism, or frequency of I/D alleles, in athletes, in comparison to controls.

Reference	Number of subjects	Training/sport	Variable assessed as indicator of superior athletic ability	Measurements remarks	Findings
[3]	33	high altitude mountaineers	number of successful climbs above 8,000 m	single measurement compared to achievement	I allele associated with improved endurance
[24]	60 Spanish athletes	cycling, running, handball	frequency of alleles in elite athletes	single measurement compared to competition result	I allele more frequent in athletes in comparison to controls
[25]	64 Olympic athletes	rowing	frequency of alleles in professional athletes	single measurement	I allele more frequent in elite rowers
[6]	91 Olympic athletes	running	frequency of alleles in elite athletes in comparison to controls	single measurement	I allele more frequent in elite runners
[15]	447 athletes	Triathlon	frequency of alleles in association with performance results	single measurement	I allele associated with best finishing times on South African born Ironman athletes
[16]	88 Turkish athletes	running	frequency of alleles in association with performance results	single measurement	D allele associated with better performance in short sprints
[19]	192 athletes	6 different modalities	frequency of alleles in athletes in comparison to controls	single measurement	No difference in the frequency of the alleles between athletes and controls
[26]	56 elite athletes	swimming	frequency of alleles in elite athletes grouped by distance	single measurement	D allele present in higher frequency in short distance swimmers
[17]	281 Kenyan athletes	long distance running	frequency of alleles in athletes in comparison to controls	single measurement	No difference in the frequency of the alleles between athletes and controls
[27]	121 elite Israeli runners	79 Marathon runners and 42 sprinters	frequency of alleles in elite athletes grouped by distance	single measurement	D allele in higher frequency in Israeli sprinters

What emerges from the analysis of the studies listed in Table 2 is that the ACE I/D polymorphism seems to imprint positive characteristics in athletes devoted to certain sport modalities, and certain ethnicities. The studies which failed to demonstrate that certain alleles were more frequent in athletes in comparison to the general population usually included various ethnicities, and multiple sports modalities were grouped together when the comparisons were made. Again it seems unlikely that the ACE gene would have a “blanket” effect in conferring superior qualities to all athletes, from all ethnicities, in whichever sport. The key to the applicability of the ACE gene in detecting better athletes seems to lie in determining in which sports, and which ethnicities, the correlation is strong enough to warrant further investigation.

Another interesting observation is that of the frequency of alleles according to the characteristics of the sport modality: In general, it appears that the I allele is more frequent in athletes competing in longer events, while the D allele seems to be seen in excess in athletes participating in short duration competitions.

Most of the publications found a positive association between the alleles in the I/D ACE polymorphism and a characteristic that allowed some athletes to be more successful than others. In some studies the use of superior performance as a variable might be arguable, since the definition of superior performance is subject to interpretation. For that reason, a better definition of improved or superior performance, or of “elite athlete”, is warranted in future studies.

The most common finding was that the D allele, and even more so the DD genotype was associated with greater ventricular hypertrophy [4,13,14], therefore contributing to better cardiac output. In relation to the I allele, the most common findings related to better outcomes in training and competition in sports such as Triathlon [15], distance running [24], and Olympic sports requiring endurance [6,25]. However, a study focusing exclusively on Kenyan athletes failed to demonstrate the association between the polymorphism and quality of the athletes [17]. 

Studies involving Olympic athletes [6,25], regardless of the sport category found that the I allele was associated with better performance, again in sports requiring endurance. The study investigating triathletes [15] also found an association between the I allele and better finishing times, although the study found marked differences regarding ethnicity of the individuals and the ACE polymorphism results. These results may reflect that the DD and II genotypes affect a variety of factors, not just through blood pressure, but have other effects on cardiac size, tissue perfusion and muscle oxygen transport, and that these differences may be partially influenced by ethnicity. It is obvious from the results concerning the I/D polymorphism of the ACE gene, as most genetic factors are, that the situation is much more complex than a linear positive correlation between a polymorphism and better performance, but that does not exclude benefits to be gained in increased knowledge about interactions between the ACE gene and environmental factors that might contribute to a more appropriate phenotype for the execution of a specific sport.

## 5. Conclusions

There is a considerable amount of information available relating to ACE and its genetic polymorphism in humans, including its potential use for identification of elite athletes. However, repeatability is lacking and the variation in study design and the population studied has prevented a final conclusion to be drawn regarding the usefulness of ACE as a biomarker of performance in human beings**.** However, there seems to be more evidence supporting the fact that ACE II polymorphism (and lower ACE activity) has a correlation with aptitude for sports requiring endurance, while the DD polymorphism (and higher ACE activity) relates to sprint ability. Similar correlations between ACE genotype and activity and improved athletic performance have been demonstrated in other species, such as horses [2]. Several paradoxes still need investigation before a definite answer regarding the value of I/D polymorphism in predicting athletic ability can be presented. These include: the effects of ethnicity, sport modality, variables influencing post-translational shedding of ACE, and the effects of local RAS in athletes. 
